# Mechanical Processing of Lipoaspirate With a Fluidic Device Platform Promotes Wound Healing Transcriptional Programs and Angiogenesis In Vitro

**DOI:** 10.1093/asj/sjaf055

**Published:** 2025-04-10

**Authors:** Jeremy A Lombardo, Derek A Banyard, David Zalazar, Mary Ziegler, Alexandria M Sorensen, Pisrut Phummirat, Alan D Widgerow, Jered B Haun

## Abstract

**Background:**

Mechanical processing of lipoaspirate (LA) produces a stromal vascular fraction (SVF) without enzymatic digestion for use in aesthetic, surgical, and regenerative applications. We recently presented novel device technologies that increased mesenchymal stem cell (MSC) content relative to standard nanofat (NF) processing.

**Objectives:**

Here, we introduce a third technology designed to enhance fluid shear forces and explore the impact of mechanical processing on regenerative potential in vitro.

**Methods:**

Human LA samples were processed with our previously reported emulsification micronization device and filtration device, and then optimized using a new shearing device (SD). Results were analyzed for total cell count, viability, and percentages of endothelial progenitor cells (EPCs) and MSCs compared to manual NF processing, both immediately and following 24-hour culture. Expression of genes related to wound healing was quantified by real-time quantitative polymerase chain reaction, and angiogenic capacity was determined with an in vitro 3-dimensional sprouting assay.

**Results:**

The SD did not significantly affect MSC recovery or viability, but EPCs were enriched in a shear stress–dependent manner. Gene expression was not altered immediately after processing, but after culture we noted changes to wound-healing transcriptional programs that were consistently stronger for our devices than NF. Differences were statistically significant for CXCL1, IL1β, IL6, CSF3, and COL1A2. Notably, angiogenic vessel sprouting was significantly enhanced for our devices compared to NF.

**Conclusions:**

Mechanical processing of lipoaspirate with our 3-device platform resulted in greater enrichment of stem and progenitor cells, activation of genes implicated in wound healing, and induction of angiogenesis in vitro relative to NF. Future studies will ascertain potential implications in vivo for all indications that currently utilize NF, as well as automate the process within an integrated system.

Adipose tissue has been established as a rich source of regenerative potential, but optimal methodologies are still being sought for processing samples and delivering cellular therapeutics to patients.^1-4^ Adipose-derived stem cells (ADSCs) have shown exciting therapeutic potential but are impractical due to the need for ex vivo culture. The stromal vascular fraction (SVF) is a heterogenous mixture of cells and connective tissue that can be directly obtained from adipose tissue without the need for purification or culture, increasing its translational potential. The safety of autologous SVF has been vetted in numerous settings, and clinical trials have been completed for treatment of knee osteoarthritis (OA).^5-7^ Early SVF protocols relied upon proteolytic digestion, which introduces foreign components and may reduce healing potential.^8^ Mechanical isolation approaches were then developed, such as repeated shuffling of lipoaspirate (LA) between 2 syringes to produce nanofat (NF), which has shown promise in cosmetic applications, fat grafting, and treatment of scars, OA, and perianal fistulae.^6,9-11^ However, different approaches have been taken to mechanically micronize LA, raising important questions about standardization.^6,12-18^

We previously established that stem and progenitor cells are enriched in NF, and then developed novel fluidic device technologies to improve processing methodology.^19,20^ The emulsification and micronization device (EMD) and a filtration device (FD) combined to increase percentage (>1.5-fold) and number (2-fold to 3-fold) of MSCs and endothelial progenitor cells (EPCs) relative to standard NF. Population enrichment extended to an MSC subset called multilineage differentiating stress-enduring (Muse) cells, which have been associated with pluripotency, and also to DPP4+/CD55+ cells, which are associated with healing in the context of diabetes.^21-25^ We hypothesized that enhanced hydrodynamic shear forces generated by mechanical processing could directly influence the regenerative potential of processed adipose tissue. Mechanotransduction has previously been shown to improve cutaneous tissue growth and repair through changes in gene expression, secretome, and proliferation of different cell types.^26-28^ Additionally, mechanical fluid shear is known to modulate endothelial cell-dependent vascular remodeling, as well as the expression of tissue and matrix metalloproteinases (MMPs) in human periodontal ligament cells.^29-32^ Fluid shear has also been shown to increase various mediators related to wound healing, angiogenesis, and immune function in MSCs and ADSCs.^33-35^

Here, we evaluate a new microfluidic technology adapted to enhance shear stresses on tissue, following processing with the EMD and FD technologies.^20,36,37^ The resulting 3-device platform (Figure 1C) is then evaluated for enrichment of stem and progenitor cells, wound healing and inflammatory responses at the transcriptomic level, and angiogenic potential with a 3-dimensional (3D) in vitro sprouting assay.

**Figure 1.**
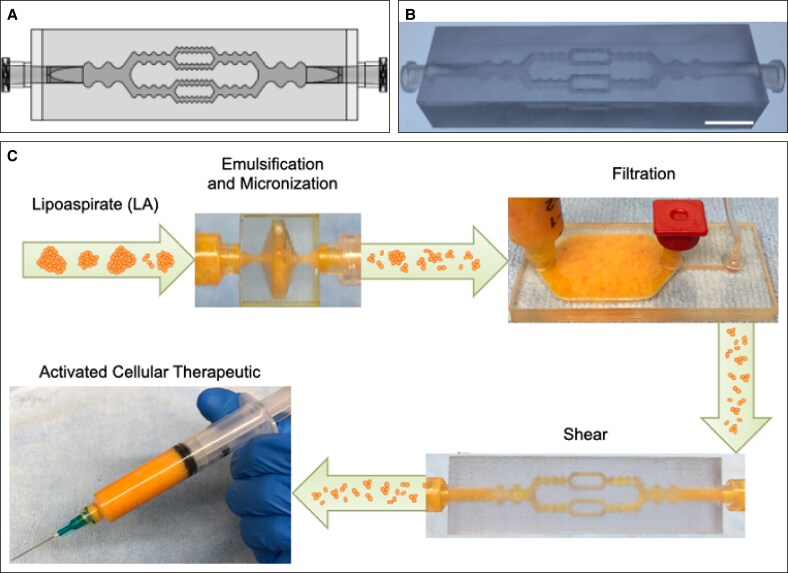
Fluidic device platform for processing human lipoaspirate. (A) Schematic of the shearing device (SD) showing branching channel architecture and repeating expansions/constrictions, both of which generate shear stresses. (B) Image of SD manufactured by 3-dimensional printing. (C) Three-device platform for mechanical processing of lipoaspirate (LA). The emulsification and micronization device (EMD) emulsifies fat droplets and also breaks down pieces of adipose tissue into smaller fragments. The filtration device (FD) then removes the largest remaining tissue pieces, and finally the SD applies additional shear stress to further break down tissue fragments and potentially activate resident stem/progenitor cellular populations. Following device platform processing, the final cell suspension can be injected directly into a patient for augmenting wound healing or for another regenerative capacity. Scale bar is 1 cm.

## METHODS

This study was conducted in accordance with the regulations of the University of California, Irvine Institutional Review Board (no. 2015-2181) and the Long Beach VA Hospital Institutional Review Board (no. 01308) and was conducted between April 2019 and April 2023. Recruited patients were undergoing liposuction for either reconstructive or cosmetic procedures and samples were obtained with informed written consent. Patients with an active systemic infection or on immunosuppressive therapy were excluded from study participation. Lipoaspirate was harvested from 14 donors by vacuum-assisted liposuction with harvest cannulas ranging in size from 3 to 5 mm. Patients primarily self-identified as Caucasian and Hispanic, female, and between the ages of 31 to 52. Male and other races including African American were represented as well. Lipoaspirate was harvested from the body from various locations, including the abdomen, flanks, mid and lower back, and waist. Following lipoaspirate collection, samples were transported to the laboratory at room temperature and immediately processed on the same day of harvest.

### Device Design, Fabrication, and Operation

The EMD and FD were described previously.^20^ The shearing device (SD) was adapted from a device previously developed for disaggregation of small cell clusters resulting from tissue dissociation.^36,37^ Features included a network of branching channels with repeated expansion and constriction regions to generate shear forces. We modified this device for use with LA that had first been processed with the EMD and FD (Figure 1A). The primary goal was to further break down tissue fragments, reducing needle clogging during injection. A hypothesized secondary goal was to enhance cell activity by mechanotransduction. Channel dimensions of the shearing device (SD) were 3, 1.5, and 0.75 mm in width for the 3 different stages. The height was 0.75 mm for each stage. The SD was 3D printed by 3D Systems (Rock Hill, SC) with biocompatible Accura ClearVue resin as a single part to withstand high flow rates and pressures experienced during operation (Figure 1B).

The SD was evaluated with patient LA (*n* = 3) that had first been processed with the EMD and FD. Devices were sterilized with 70% ethanol and dried, and all processing steps were performed under aseptic methods in a laminar flow hood. LA was first washed with phosphate-buffered saline (PBS). The washed LA was then subdivided into separate portions. One portion was utilized directly and termed macrofat (MF). A second portion was processed under optimal conditions with the EMD (30 passes at 20 mL/s) and the FD (single pass at 10 mL/min).^20^ The remaining sample was processed with the EMD and FD as described above, followed by the SD for 20 passes at a 1.7, 5, or 15 mL/s flow rate. For all devices, fluid flow was driven by a high-precision syringe pump to ensure accurate (within 0.35% of set rate) and reproducible (within 0.05% of actual rate) performance.

### Cell Analysis

Single cells were released with collagenase and analyzed as previously described.^20^ LA was mixed with 0.1% type I collagenase (Sigma-Aldrich Co., St. Louis, MO) at a 1:1 volume ratio, incubated at 37°C for 30 minutes while swirling intermittently, separated by gravity for 10 minutes, and the infranatant layer containing SVF was collected. Recovered sample was then filtered through a 100-μm cell strainer, centrifuged at 500×g for 7 minutes, and resuspended in control media (DMEM supplemented with 500 μg streptomycin, 500 IU penicillin, and 10% fetal bovine serum). Nucleated cell counts and viability were determined with an automated, dual-fluorescence cell counter (Logos Biosystems Inc., Annandale, VA). Cell subtypes were then identified by flow cytometry, as previously reported.^20^ Cells were stained with monoclonal antibodies (all from BioLegend, San Diego, CA) specific for CD34 (clone 561, BV421 conjugate), CD45 (clone 2D1, BV510 conjugate), SSEA-3 (clone MC-631, FITC conjugate), CD26 (clone BA5b, PE conjugate), CD31 (clone WM59, PE/Cy7 conjugate), CD55 (clone JS11, APC conjugate), and CD13 (clone WM15, APC/Cy7 conjugate) in PBS with 1% BSA (PBS+) for 20 minutes at 4°C, washed by centrifugation, and then resuspended in PBS+ supplemented with 7-AAD (BD Biosciences, San Jose, CA) for dead cell exclusion. Stained cells were then analyzed with a NovoCyte 3000 Flow Cytometer (ACEA Biosciences, San Diego, CA). Signal positivity was determined with appropriate fluorescence minus one (FMO) controls and compensated data was analyzed with FlowJo software (Ashland, OR).

### Cell Culture

LA from patients (*n* = 3) was mechanically processed as described above with the EMD and FD alone, or the EMD/FD and the SD at 15 mL/s flow rate. Controls included MF as well as NF that was processed by manually passing LA 30 times between 2 connected syringes and then filtering with 1-mm mesh cloth, as described by Tonnard et al.^9^ All samples were mixed with control media at a 1:1 volume ratio, plated in petri dishes, and incubated for 24 hours at 37°C with 5% CO_2_ to allow for investigation of longer-term cell viability and transcriptional changes. Following culture, samples were digested with collagenase and then assessed for total cell number, cell viability, and stem/progenitor cell composition, as described above.

For use in a 3D in vitro angiogenesis assay, human umbilical vein endothelial cells (HUVECs) were purchased from Lonza (Bend, OR) and grown in endothelial growth medium-2 (Lonza) with 1% penicillin and streptomycin (Corning Cellgro; Thomas Scientific, Swedesboro, NJ).

### Transcriptional Analysis by RT-qPCR

Changes in gene transcription were determined for MF, NF, EMD/FD, and EMD/FD plus SD at 15 mL/s conditions (*n* = 4) with a human wound-healing RT2 Profiler PCR Array (Qiagen, Germantown, MD) immediately after processing (t = 0) and after 24 hours in culture. RNA was extracted by adding TRIzol reagent (Invitrogen, Carlsbad, CA) at a 1:1 ratio in Lysing Matrix D tubes with ceramic beads (MP Biomedicals, Irvine, CA). A FastPrep-24 instrument (MP Biomedicals) was employed to homogenize the sample for 40 seconds at 6 m/s. Samples were then snap frozen in liquid nitrogen. Before analysis, chloroform was added to the organic layer. After phase separation, RNA was precipitated from the aqueous layer with isopropanol and washed with 75% ethanol. RNA was then resuspended in RNase-free water and purified with an RNeasy mini kit with on-column DNase digestion (Qiagen). RNA quality and concentration were assessed with a NanoDrop ND-1000 (Thermo Fisher, Waltham, MA). Extracted RNA was reverse transcribed into cDNA with an RT2 First Strand kit (Qiagen) and analyzed on an Mx3005P qPCR system (Agilent Technologies, Santa Clara, CA) following manufacturer instructions. Gene expression results for each sample were normalized to a reference gene, RPLP0, which was most consistent between the 0-hour and 24-hour time points (Appendix, Supplemental Figure 1, located online at https://doi.org/10.1093/asj/sjaf055). Results for NF, EMD/FD, and SD conditions were normalized to MF at the same time point. Additionally, MF was normalized between the 0-hour and 24-hour time points.

### 3D in Vitro Angiogenesis Assay

Methods were adapted from previous work.^38^ HUVECs were coated onto collagen-coated microcarrier beads (Sigma) and embedded into a fibrin gel (MP Biochemicals). Endothelial cell growth medium containing SVF (MF digested with collagenase and washed by centrifugation) was the control. For experimental conditions, mechanically processed LA from NF, EMD, EMD/FD, or EMD/FD + SD protocols were directly added to the wells. Cultures were maintained for 10 days, with imaging by light microscopy performed on Days 1, 4, 6, and 10. For quantification, 30 beads from each condition were selected randomly, and the number of sprouts per bead was counted manually for each experiment (*n* = 4). Sprouts were only counted if they were at least half of the bead diameter.

### Statistics

Data were presented as the mean ± standard error from at least 3 independent patient samples, after normalization to the MF treatment from the same patient sample. Statistical analysis was performed with Minitab software (State College, PA) with 1-way analysis of variance (ANOVA) with Tukey's post-hoc test or the *t* test. Differences were considered statistically significant for *P* values < .05.

## RESULTS

### Shearing Device (SD) Enriches EPCs

**Figure 2.**
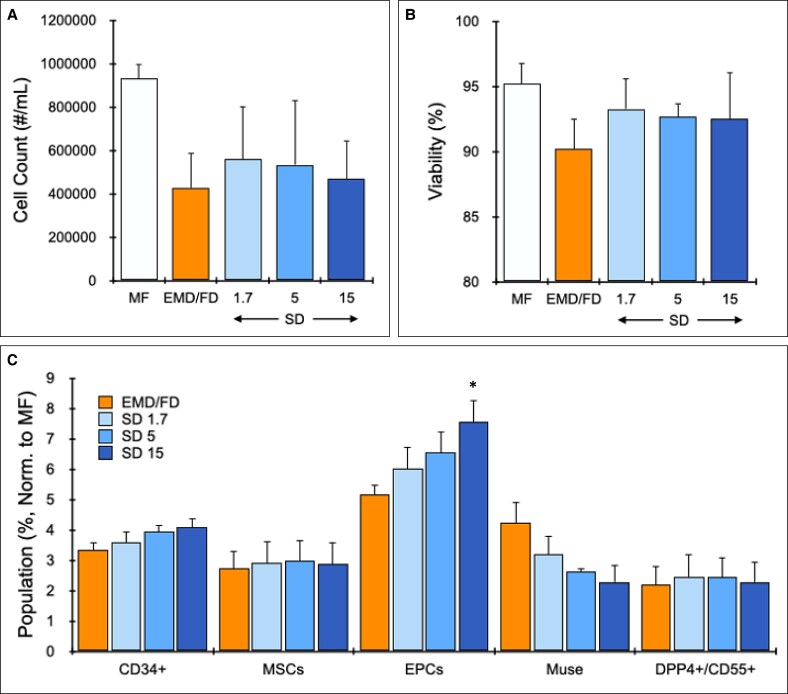
Shearing device (SD) results. LA (lipoaspirate) from patients (*n* = 3) was mechanically processed with both the EMD (emulsification and micronization device) and FD (filtration device). Samples were then processed with the SD for 20 passes at a 1.7, 5, or 15 mL/s flow rate. All samples were collagenase-digested before cellular analysis. (A) Nucleated cell counts decreased by approximately half for all device conditions in comparison to MF (macrofat). (B) Nucleated cell viability for all conditions remained at ∼90%. (C) Stem/progenitor cell populations were characterized with flow cytometry, and the relative population percentages were calculated and normalized to MF (value = 1). Processing with the SD increased EPCs (endothelial progenitor cells) in a dose-dependent manner with flow rate. MSC (mesenchymal stem cell) subpopulations were largely unaffected, but Muse (multilineage differentiating stress-enduring) cells decreased slightly with flow rate. Error bars represent standard errors from at least 3 independent experiments. Asterisk (*) indicates *P* < .05 relative to EMD/FD.

The SD was tested under different flow rates with human LA specimens (*n* = 3) and compared to MF and EMD/FD processing alone. Total cell counts were highest for MF at 930,000 +/− 60,000 cells/mL LA sample and decreased to less than half with EMD/FD treatment at 430,000 +/− 160,000 cells/mL LA (Figure 2A). These values were comparable to previous results in both cases.^20^ Additional treatment with the SD did not affect total cell count at any flow rate, remaining comparable to the EMD/FD condition. Cell viability remained at >90% for all conditions (Figure 2B). Total cell count and viability differences between groups were not statistically significant. The relative percentage of MSC and EPC subpopulations were determined with flow cytometry; results are presented in Figure 2C after normalization to MF (value = 1). All stem and progenitor cell populations were enriched by at least 2-fold for all device conditions. SD processing modestly enhanced CD34 + cell percentage in a dose-dependent manner with flow rate. Overall, CD34 + cells increased from ∼3.3-fold higher percentage than MF for EMD/FD alone to ∼4-fold for SD at a 15 mL/s flow rate, but differences were not statistically significant. MSCs were not affected by SD treatment, remaining at 3-fold higher percentage than MF for all device processing conditions. The SD had a stronger effect on EPCs, however, increasing in a dose-dependent manner from ∼5-fold greater percentage than MF for EMD/FD alone to 7.6-fold for SD at the 15 mL/s flow rate. Importantly, the difference between EMD/FD and SD at 15 mL/s was statistically significant (*P* = .035). Interestingly, Muse cell percentage decreased with the SD in a dose-dependent manner with flow rate, but the 15 mL/s flow rate condition remained at a >2-fold higher percentage than MF. DPP4+/CD55+ cell populations were unaffected by SD processing, remaining at ∼2.5-fold higher percentage than MF. Population percentages without normalization to MF were similar (Appendix, Supplemental Figure 2, located online at https://doi.org/10.1093/asj/sjaf055). Variability was high for MSCs, most likely due to different baseline values across patients and/or anatomical locations of adipose tissue harvest.^39,40^ However, EPC percentages were more consistent, and all device conditions were statistically different compared to MF (*P* < .01).

### Transcriptomic Analysis of Wound Healing

Quantitative reverse transcription polymerase chain reaction (RT-qPCR) was performed with a wound healing panel for MF, NF, EMD/FD, and EMD/FD plus SD at 15 mL/s conditions immediately after processing (*n* = 4). Expression for each gene was normalized to the housekeeping gene RPLP0 (Appendix, Supplemental Figure 3, located online at https://doi.org/10.1093/asj/sjaf055), and then mechanical processing results (NF, EMD/FD, SD) were normalized to MF. Changes in gene expression were modest across the different processing methods, varying by at most a factor of 2 (Appendix, Supplemental Figure 1). Compared to NF, the only difference approaching significance was for the gene PLG with SD processing. Normalized expression of PLG dropped to 0.18 +/− 0.09 for NF, but increased to 1.77 +/− 0.34 for SD (*P* = .052). PLG was also downregulated for EMD/FD to 0.39 +/− 0.34, but this difference was also not significant. However, expression changes were generally similar for all mechanical processing methods.

We also evaluated cells after being cultured for 24 hours, which would provide time for transcriptional changes to occur following any activation stimulus. Following culture, cell numbers for the EMD/FD and SD conditions were similar to before processing, at 340,000 +/− 150,000 and 380,000 +/− 180,000 cells/mL LA, respectively, whereas MF decreased by almost one-third, to 610,000 +/− 130,000 cells/mL LA (Appendix, Supplemental Figure 4, located online at https://doi.org/10.1093/asj/sjaf055). NF was the lowest at 240,000 +/− 100,000 cells/mL LA, but differences were not statistically significant for any test condition. Trends for viability and percentage of stem/progenitor cell subpopulations were similar to those observed before culture (Appendix, Supplemental Figure 4), aside from modest enrichment of Muse and DPP4+/CD55 + cells. Population percentages without normalization to MF demonstrated similar trends (Appendix, Supplemental Figure 5, located online at https://doi.org/10.1093/asj/sjaf055).

**Figure 3.**
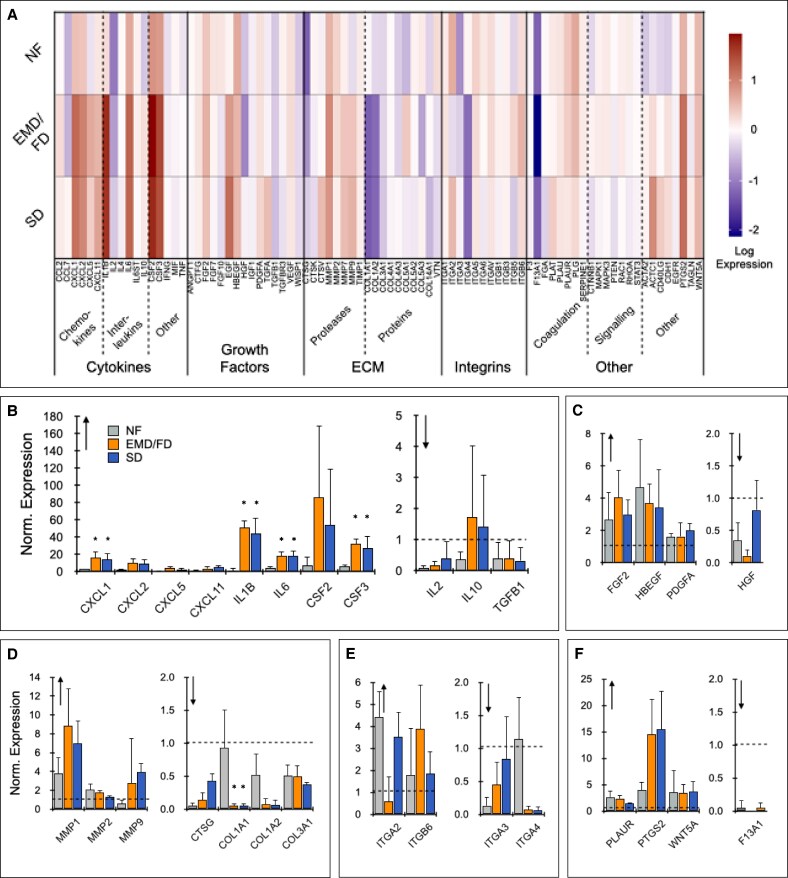
Gene expression after 24-hour culture. LA (lipoaspirate) from patients (*n* = 4) was processed into either NF (nanofat), with the EMD (emulsification and micronization device) and FD (filtration device), or with the EMD/FD followed by SD (shearing device) at 15 mL/s. RNA was extracted and RT-qPCR (quantitative reverse transcription polymerase chain reaction) was performed with a wound-healing panel, and results were normalized to MF (macrofat; value = 1). (A) Heat map for all wound healing–related genes. (B-F) Normalized expression for genes in which at least 1 mechanical processing condition was upregulated or downregulated by >50% relative to MF. Genes are grouped as (B) cytokines, (C) growth factors, (D) extracellular matrix, (E) integrins, and (F) other. Error bars represent standard errors from at least 3 independent experiments. Asterisk (*) indicates *P* < .05 relative to NF.

Gene expression by RT-qPCR after 24-hour culture (*n* = 4) is shown in Figure 3A. Mechanical processing elicited stronger changes in gene expression, particularly for genes related to inflammation, angiogenesis, and matrix remodeling. All changes of at least 2-fold relative to MF are presented as bar graphs in Figure 3B-F. Overall, we observed that all 3 mechanical processing treatments caused similar changes in gene expression, but to varying magnitudes.

The chemokines CXCL1 and CXCL2 (MIP2-α) and cytokines IL-1β, IL-6, CSF2 (GM-CSF), and CSF3 (G-CSF), were all upregulated over MF by >10-fold for EMD/FD and SD processing (Figure 3B). These genes were also upregulated for NF, but more modestly, between 4-fold and 10-fold over MF. Differences were statistically different between NF and EMD/FD or SD for CXCL1 (*P* = .017 EMD/FD vs NF; *P* = .034 SD vs NF), IL-1β (*P* = .018 EMD/FD vs NF; *P* = .023 SD vs NF), IL-6 (*P* = .011 EMD/FD vs NF; *P* = .013 SD vs NF), and CSF3 (*P* = .028 EMD/FD vs NF; *P* = .049 SD vs NF). Moreover, CXCL5 and CXCL11 increased for EMD/FD and SD treatments, but not NF, although differences were not statistically significant. IL-2 and TFG-β1 were downregulated for each mechanical processing condition. The EMD/FD and SD produced very similar expression results for these genes, and differences were not statistically significant. We also noted that CXCL2, CXCL5, and CXCL11 were all modestly elevated before culture (Appendix, Supplemental Figure 1, located online at https://doi.org/10.1093/asj/sjaf055).

Growth factors FGF2 (basic FGF), HBEGF (heparin-binding EGF-like growth factor), and PDGFA (platelet-derived growth factor subunit A) were upregulated after culture for each mechanical processing condition, although at more modest levels of ∼1.5-fold to 4-fold (Figure 3C). HGF was downregulated for each condition, but most strongly for EMD/FD (Figure 3C). PDGFA was also elevated before culture (Appendix, Supplemental Figure 1), with NF providing the strongest effect.

MMPs 1, 2, and 9 were all upregulated after 24-hour culture, with MMPs 1 and 9 substantially higher for EMD/FD and SD than for NF, although differences were not statistically significant (Figure 3D). MMP9 was also consistently upregulated before culture (Appendix, Supplemental Figure 1). We noted a decrease in expression for genes that encode for collagen synthesis, including COL1A1, COL1A2, and COL3A1. Effects were stronger for EMD/FD and SD than for NF; differences were statistically significant for COL1A1 (*P* = .041 EMD/FD vs NF; *P* = .034 SD vs NF). Integrin subunits α2 and β6 increased, whereas subunits α3 and α4 decreased, but all showed high variability (Figure 3E). PTGS2 (COX-2) expression increased by >10-fold for EMD/FD and SD treatments after culture, whereas NF elicited a more modest 3-fold increase, but differences were not statistically significant (Figure 3F).

Finally, we noted that transcriptional changes did take place in MF between the initial time point and after 24 hours of culture (Appendix, Supplemental Figure 6, located online at https://doi.org/10.1093/asj/sjaf055). Changes included CXCL1 and CXCL5 expression, suggesting that mechanical shear provided further upregulation. The chemokines CCL2 and CC7 increased expression with culture only, but these genes appeared to be mechanically insensitive. Additionally, MMP1 and the integrin subunits, α2, α5, and β3 increased with MF culture, whereas several COL family genes and integrin subunits α2, α4, α6, β1, and β6 decreased.

### 3D in Vitro Angiogenesis Assay

A 3D angiogenesis assay was performed to assess how device-processed LA stimulated endothelial sprouting and vessel formation in vitro in comparison to collagenase-digested MF (SVF) and NF (*n* = 4). Device conditions included EMD, EMD/FD, and EMD plus SD at 15 mL/s. At Day 1, all device conditions had initiated modest tip cell formation (Appendix, Supplemental Figure 7, located online at https://doi.org/10.1093/asj/sjaf055). Conversely, SVF and NF exhibited hypersprouting and even some branching. By Days 4 and 6, NF sprouts were almost completely disconnected (Appendix, Supplemental Figure 7). SVF and the device conditions all displayed increased sprouting and proliferation, but also some loss of connections (ie, wandering sprouts).

**Figure 4.**
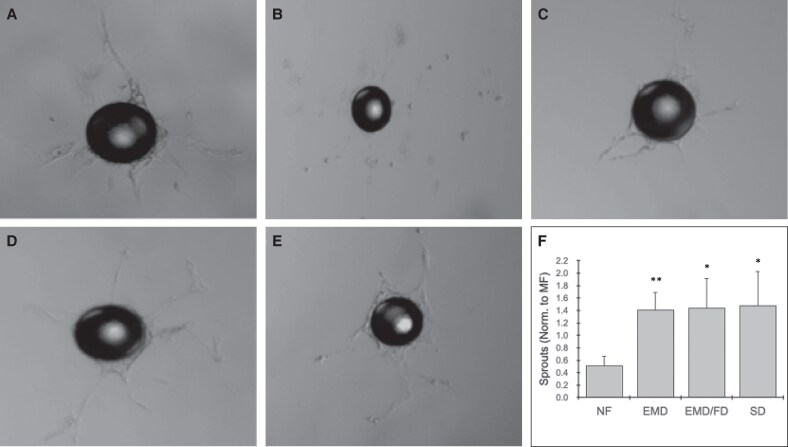
Mechanical processing with device platform supports sprouting angiogenesis. HUVECs were coated onto collagen beds, embedded into a fibrin gel, and then co-cultured with human LA (lipoaspirate; *n* = 4) that was (A) enzyme-digested MF (macrofat; SVF, or stromal vascular fraction); (B) NF (nanofat; or SVF processed from (C), (D), or (E). (C) EMD (emulsification and micronization device); (D) the EMD and FD (filtration device); (E) EMD/FD + SD (shearing device). These are representative images for each condition on Day 10 of culture. (F) For each condition, sprouts were manually counted from 30 beads, averaged, and normalized to MF. Error bars represent standard errors from at least 4 independent experiments. * and ** indicate *P* < .05 and .01, respectively, relative to NF.

At Day 10, SVF retained evidence of connected sprouts (Figure 4A). In contrast, NF showed only previously formed sprouts that were no longer connected (Figure 4B). The device-processed samples (Figure 4C–E) were all generally similar to SVF. However, each device condition produced higher average numbers of sprouts per bead compared to SVF, by ∼40% (Figure 4F). Sprout numbers for NF were ∼2-fold lower relative to SVF, and almost 3-fold lower relative to the device conditions. Sprout number differences between the device conditions and NF were all statistically significant (*P* = .009 for EMD, *P* = .03 for EMD/FD, and *P* = .04 for EMD/FD + SD).

## DISCUSSION

The new shearing device (SD) did not negatively impact overall cell recovery or viability, suggesting that the majority of cells within LA can withstand the enhanced shear forces generated during this additional processing step. Importantly, flow cytometric analysis revealed a shear-dependent enrichment of EPCs, which may impact angiogenesis and tissue regeneration.^41^ Total MSCs, as well as the DPP4+/CD55 + subpopulation important for wound healing in the context of diabetes, remained constant.^25^ Muse cells did appear to decrease with shear, an interesting finding given that this MSC subpopulation is considered to be stress-tolerant, at least under conditions such as hypoxia, low temperature, prolonged collagenase exposure, and serum deprivation.^21^ Overall findings were largely maintained after 24 hours in culture, suggesting that stem/progenitor cell subpopulations can survive longer term after mechanical processing. Total cells decreased by 30% for MF, but EMD/FD and SD conditions only decreased by 15% to 20%. It is possible that mechanical treatment may select for cells that are hardier, but if this were the case, this selection extended to most cell types and not just stem/progenitors. Manual NF processing produced fewer total cells and lower relative numbers of stem/progenitor cells, as we previously observed.^20^

Changes in gene expression related to wound healing were modest immediately after processing, and likely reflected changes in cellular composition because there was insufficient time for transcriptional response. Considerably larger changes were observed after 24-hour culture that may have been impacted by mechanotransduction. Mechanical fluid shear has previously been linked to elevated gene expression and/or secretion for many of the genes that were most strongly upregulated, including IL-1β, IL-6, CSF2, CSF3, CXCL1, CXCL2, PTGS2, HBEGF, FGF2, and MMP1.^29,30,33,34,42-46^ The mechanical fluid shear response of CSF3 was specifically observed in ADSCs.

We also observed evidence that fluidic processing promoted angiogenic transcriptional programs. FGF2 and PDGFA expression increased more than 2-fold for all mechanical treatments, and both strongly promote angiogenesis by increased cell recruitment and proliferation.^47^ We did observe a decrease in the angiogenic factor HGF. Additional genes have been shown to increase cell recruitment, proliferation, tube formation, and induction of proangiogenic factors, including MMPs 1, 2, and 9, as well as inflammatory mediators CXCL1 and CXCL2, CSF3, IL-1β, and PTGS2.^48-56^ Combined with EPC enrichment demonstrated by flow cytometry, these results suggest that mechanical processing of LA should enhance wound healing and/or fat graft survival through neovascularization.

Indeed, the 3D in vitro angiogenesis assay confirmed that mechanical processing LA with the devices produced significantly more sprouting than NF, and with better connection maintained throughout the 10-day time course. Although NF induced a strong initial response, endothelial cells disconnected from sprouts by Day 4. Overexpression of the Slug transcription factor in endothelial cells has been associated with excessive sprouting and sprout wandering.^57^ SVF also displayed strong initial sprouting that could be linked to the same mechanism, but improved stabilization of sprouts long term indicates modulation in activation or secondary influences, possibly an inhibitor. Mechanical processing with the devices did not exhibit excessive initial sprouting like NF and SVF, but did display wandering similar to SVF. However, higher sprout numbers seen for the device conditions conclusively demonstrated improved angiogenic potential. Interestingly, results were very similar for EMD, EMD/FD, and EMD/FD + SD cases, indicating that activation effects likely arise from the EMD, which corroborates RT-qPCR results. Further studies will be needed to enhance these effects and better stabilize formed sprouts, including investigation into Slug and Notch signaling.^58,59^

Mechanical processing also appeared to stimulate epithelial growth and matrix remodeling. Enhancements were observed for HBEGF, which accelerates re-epithelization in cutaneous wounds.^60,61^ Moreover, MMPs 1, 2, and 9 all increased, and are known to play essential roles in matrix remodeling and re-epithelialization in both acute and chronic wounds.^50,62^ Interestingly, we also noted a general decrease in collagen synthesis in the COL family, particularly COL1A1, COL1A2, and COL3A1. We acknowledge that deficiency in COL genes has been associated with impaired wound healing in murine models.^63,64^ However, overexpression has also been implicated in fibrosis and hypertrophic scars.^65-69^ Mechanical processing enhanced the α2 integrin subunit before and after 24-hour culture. The α2 subunit pairs with β1 in epithelial cells, endothelial cells, fibroblasts, and MSCs, and exerts numerous effects including binding to collagen (types 1 and 3) and MMP1, and also plays a role in wound healing through migration, angiogenesis, and collagen compaction/polymerization.^70,71^ The β6 subunit increased only with mechanical processing after culture, and is linked to proliferation and remodeling by means of αVβ6 expression in epithelial cells.^71,72^ Last, the α4 subunit greatly decreased for the EMD/FD and SD conditions after 24-hour culture, but not NF. The α4 subunit associates with β1 and β7 subunits, is expressed primarily on leukocytes, and binds to fibronectin and vascular homing receptors (VCAM-1, MAdCAM-1). Interestingly, α4 blockage is actively being pursued to ameliorate tissue damage in the context of several autoimmune diseases, predominantly acting through T cells.^73^

The most striking result was that hydrodynamic shear stress elicited a strongly proinflammatory environment (e.g., CXCL family members 1, 2, 5, 11; IL-1β; IL-6; CSF2; CSF3; and PTGS2). This was observed for NF but was substantially heightened by the fluidic device processing platform. Notably, differences between the device conditions and NF were statistically significant for CXCL1, IL-1β, IL-6, and CSF3. Inflammation is invariably associated with the early stages of healing to clear away damaged and dead tissue, as well as with influencing the immunomodulatory, regenerative, angiogenic, and anti-apoptotic potential of MSCs.^74,75^ Specifically, stimulating inflammation has been postulated as a means to enhance wound healing, and the primary mechanism has been linked to paracrine effects that enhance MSC glycolysis.^74-76^ “Priming” MSCs with inflammatory challenges like IL-1β has been shown to induce an anti-inflammatory, pro-regenerative phenotype that enhances tissue remodeling and angiogenesis.^76,77^ Furthermore, 3D culture of MSC spheroids has been shown to increase glycolysis and induce IL-1β expression, which initiates an “auto-priming” effect.^76,78,79^ However, the balance between inflammation and wound healing is delicate, and care must be given to avoid degenerating into chronic wound settings. In this case, priming would have been caused by a mechanical stimulus, not chemical, although both approaches do converge on IL-1β and potential downstream interplay of neutrophils and macrophages at the site of healing.^80,81^ Notably, this increase in inflammatory markers correlated with improved angiogenesis in the 3D sprouting model when compared to NF. We do not believe the above results were caused by an immune response to the EMD, FD, or SD device materials or microbial contamination, because biocompatible materials, sterilization, and aseptic methods were utilized for all studies. Importantly, classic mediators of inflammation such as TNF-α, CCL2 (MCP-1), and CCL7 (MCP-3) were not affected. Instead, we contend that the fluidic platform was more effective at mechanically activating cellular populations (relative to NF), leading to enhanced angiogenesis and wound healing–related gene expression.

Ultimately, this fluidic device platform will improve standardization of mechanical SVF processing and increase stem/progenitor cell enrichment, which may pave the way for more efficacious autologous cell-based aesthetic treatments, surgical grafts by cell-assisted lipotransfer, and regenerative therapeutics. Specifically, this device platform can replace nanofat and other treatments in which SVF is currently employed. This includes filtration through our device, and thus we expect that the final product can similarly be injected by small-gauge needles. Furthermore, these devices offer high potential for automation within a single instrument, which would enable high throughput fat processing and alleviate the burden on the surgeon and/or operating room staff so that they can focus on more critical portions of the procedure and the well-being of the patient.

The enhanced shear stresses from the SD did enrich EPCs in a significant manner, but other metrics remained nearly identical to the EMD and FD alone. This suggests that the mechanical effects resulting from the EMD are largely sufficient, but we will follow up with additional in vivo studies in future work.

A limitation of this study is that we did not determine which cell subtypes within the complex SVF milieus may be responding to mechanical stimuli and/or secreting factors that may influence angiogenesis and/or inflammation. This could be addressed with cell-based techniques such as single-cell RNA sequencing, which we will explore in future work. Furthermore, in this study we did not investigate cell enrichment and phenotypic changes over longer periods of time, or how they may translate to wound healing in vivo, which will also be examined in future work. Studying cell response dynamics, both in vitro and in vivo, could provide key insight into the transition from inflammation to healing and direct evidence of matrix remodeling and angiogenesis. It is also possible that the acellular components within mechanically processed SVF may contribute in part to regenerative properties, which will be studied in future work as well. We also plan to commercialize the devices as part of an instrument for clinical settings. Ultimately, this clinical platform would produce an improved NF product with minimal burden for the surgeon and operating room staff while also generating optimal benefit for the patient.

## CONCLUSIONS

We have demonstrated that mechanical processing of LA by fluid shear stress enriches MSC and EPC populations and induces unique wound-healing transcriptional programs. The new SD was designed to enhance shear stresses and seamlessly combine with the EMD and FD technologies to comprise an automated fluidic processing platform to reduce user variability and improve patient outcomes. The devices were superior to NF for stem and progenitor cell enrichment, as well as transcriptional response for genes related to wound healing and angiogenesis.

## Supplemental Material

This article contains supplemental material located online at https://doi.org/10.1093/asj/sjaf055.

## Supplementary Material

sjaf055_Supplementary_Data
