# Supplementary material for: Mechanical Processing of Lipoaspirate With a Fluidic Device Platform Promotes Wound Healing Transcriptional Programs and Angiogenesis In Vitro
Source: Aesthet Surg J. 2025 Apr 10;45(8):850–9. doi: 10.1093/asj/sjaf055 (PMC12260377; doi:10.1093/asj/sjaf055)
Supplement: sjaf055_Supplementary_Data [file sjaf055_supplementary_data.pdf]

Supplemental Information

“Mechanical processing of lipoaspirate with a fluidic device platform promotes wound healing transcriptional programs and angiogenesis *in vitro*”

Author list: Jeremy A. **Lombardo**, PhD; Derek A. **Banyard**, MD, MBA, MS; David **Zalazar**, MS; Mary **Zeigler**, PhD; Alexandria M. **Sorensen**, BS; Pisrut **Phummirat**, MS; Alan D. **Widgerow** MBBCh, MMed; and Jered B. **Haun**, PhD

RESULTS

Gene expression immediately after processing

Gene expression determined by RT-qPCR immediately after processing is shown as a heat map in Supplemental Figure 1A. Genes in which at least one of the mechanical processing conditions was 50% higher or lower than MF are shown in Supplemental Figure 1B-F. Compared to NF, the only difference approaching significance was for the gene PLG with SD processing. Normalized expression of PLG dropped to 0.18 +/- 0.09 for NF, but increased to 1.77 +/- 0.34 for SD (p = 0.052). PLG was also downregulated for EMD/FD to 0.39 +/- 0.34, but this difference was also not significant.

Effect of culture on cell number and viability

We studied how recovered cell subpopulations change with time following mechanical processing. LA specimen from patients (N=3) was processed with the EMD/FD alone, or EMD/FD plus the SD at 15 mL/s, and cultured for 24 hours. MF and manual NF processing were included as controls. After the 24-hour incubation period, total cell numbers for MF decreased by one-third from before culture, to 610,000 +/- 130,000 cells/mL LA (Supplemental Figure 4). However, EMD/FD and SD conditions remained close to pre-culture values at 340,000 +/- 150,000 and 380,000 +/- 180,000 cells/mL LA, respectively. NF was the lowest at 240,000 +/- 100,000 cells/mL LA, but differences were not statistically significant for any test condition. Trends for viability and percentage of stem/progenitor cell subpopulations were similar to those observed before culture (Supplemental Figure 4). Notable differences were that Muse and DPP4+/CD55+ cell subpopulations percentages were relatively higher and SD treatment no longer decreased Muse cells. In addition to yielding lower total cells, NF also produced lower percentages of each cell subpopulation in comparison to EMD/FD and SD treatments, but differences were not statistically significant due to high variability after culture. Population percentages without normalization to MF demonstrated similar trends (Supplemental Figure 5).

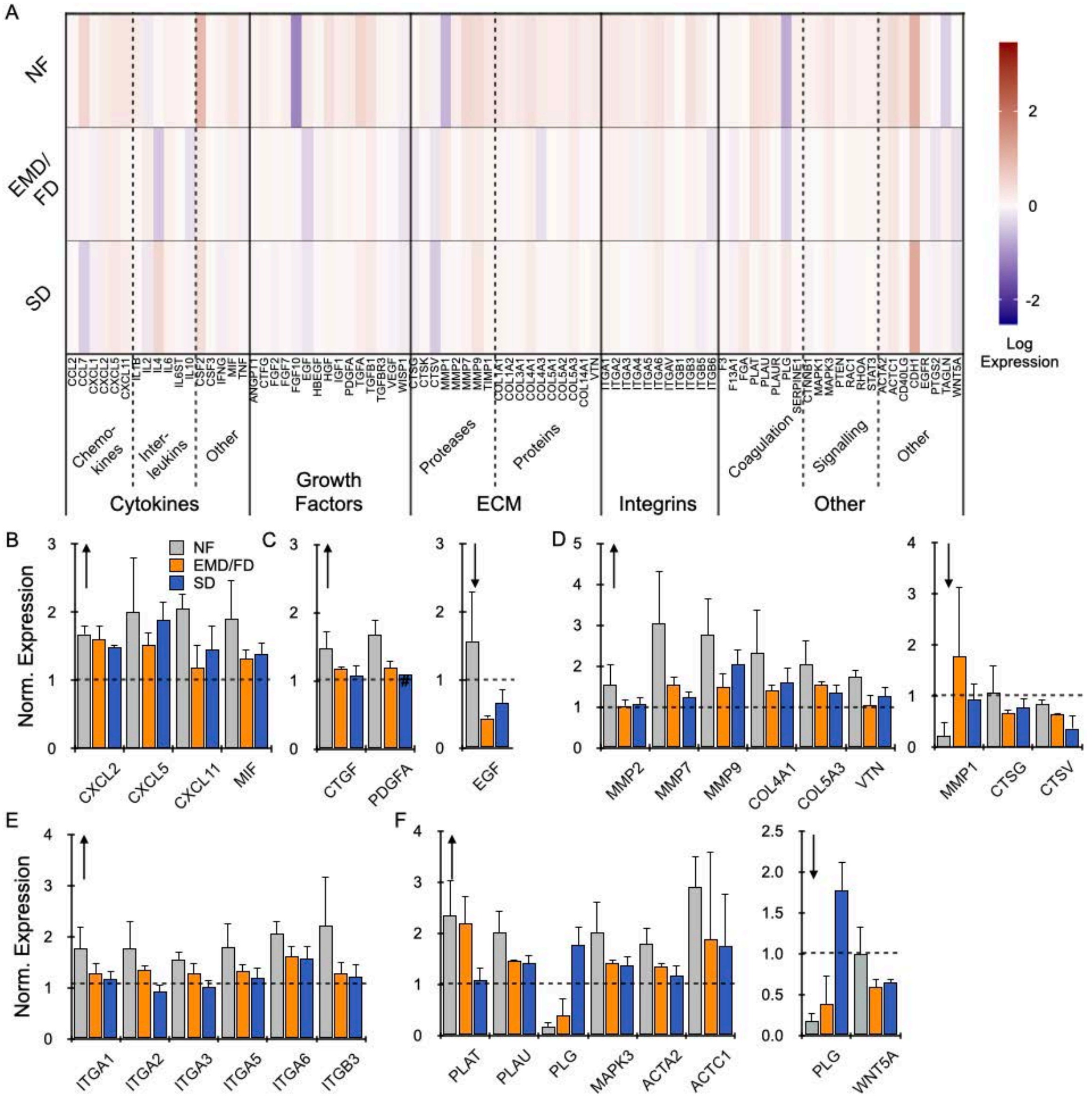

Supplemental Figure 1. Gene expression immediately after processing. Human LA (N=4) was processed as NF, with the EMD and FD (EMD/FD), or with the EMD/FD followed by SD at 15 mL/s. RNA was extracted and RT-qPCR was performed using a wound healing panel, and results were normalized to MF (value=1). (A) Heat map of all wound healing-related genes. (B-F) Results for genes in which at least one mechanical processing condition was upregulated or downregulated by >50% relative to MF. Genes are grouped as (B) cytokines, (C) growth factors (D) extracellular matrix, (E) integrins, and (F) other. Error bars represent standard errors from at least three independent experiments.

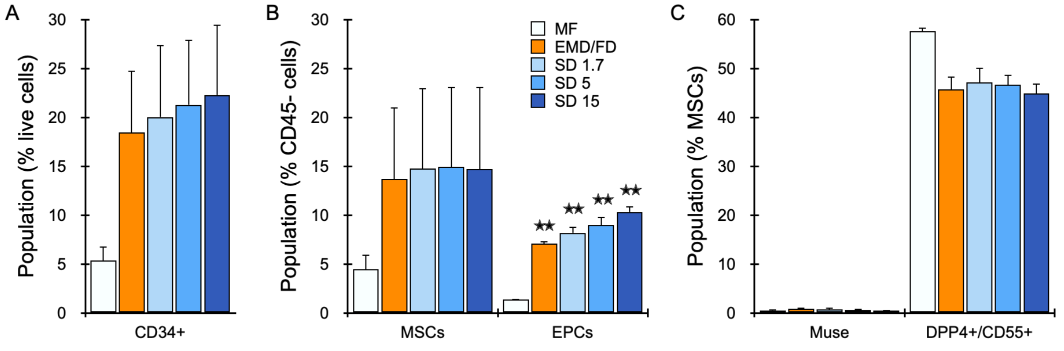

Supplemental Figure 2. Shearing Device (SD) subpopulation results without normalization. The results from Fig. 2C in the main text are presented without normalizing to MF. Population percentages are shown separately for (A) CD34+ cells, (B) MSCs and EPCs, and (C) Muse and DPP4+/CD55+ cells. Error bars represent standard error from at least three independent experiments. * indicates $p < 0.05$ and ** indicates $p < 0.01$ relative to MF.

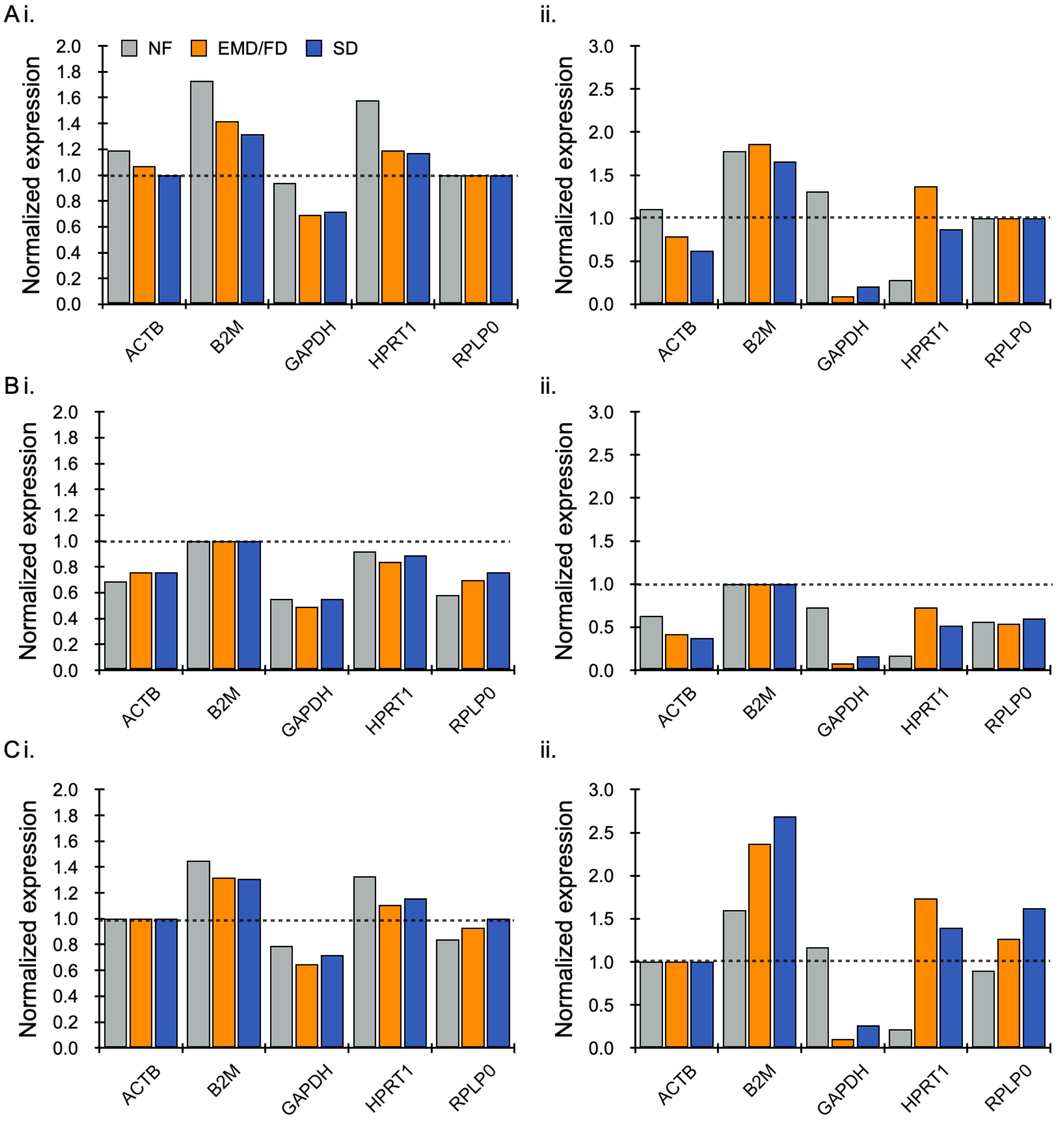

Supplemental Figure 3. Expression of house-keeping genes. RT-qPCR results for 5 house-keeping genes after normalizing to (A) RLP0, (B) B2M, and (C) ACTB values determined for macrofat (MF). Samples were measured (i) immediately after processing and (ii) after 24 culture. GAPDH and HPRT1 were not detected for some conditions and were therefore eliminated from consideration. RPLP0 displayed the most stable results at both time points.

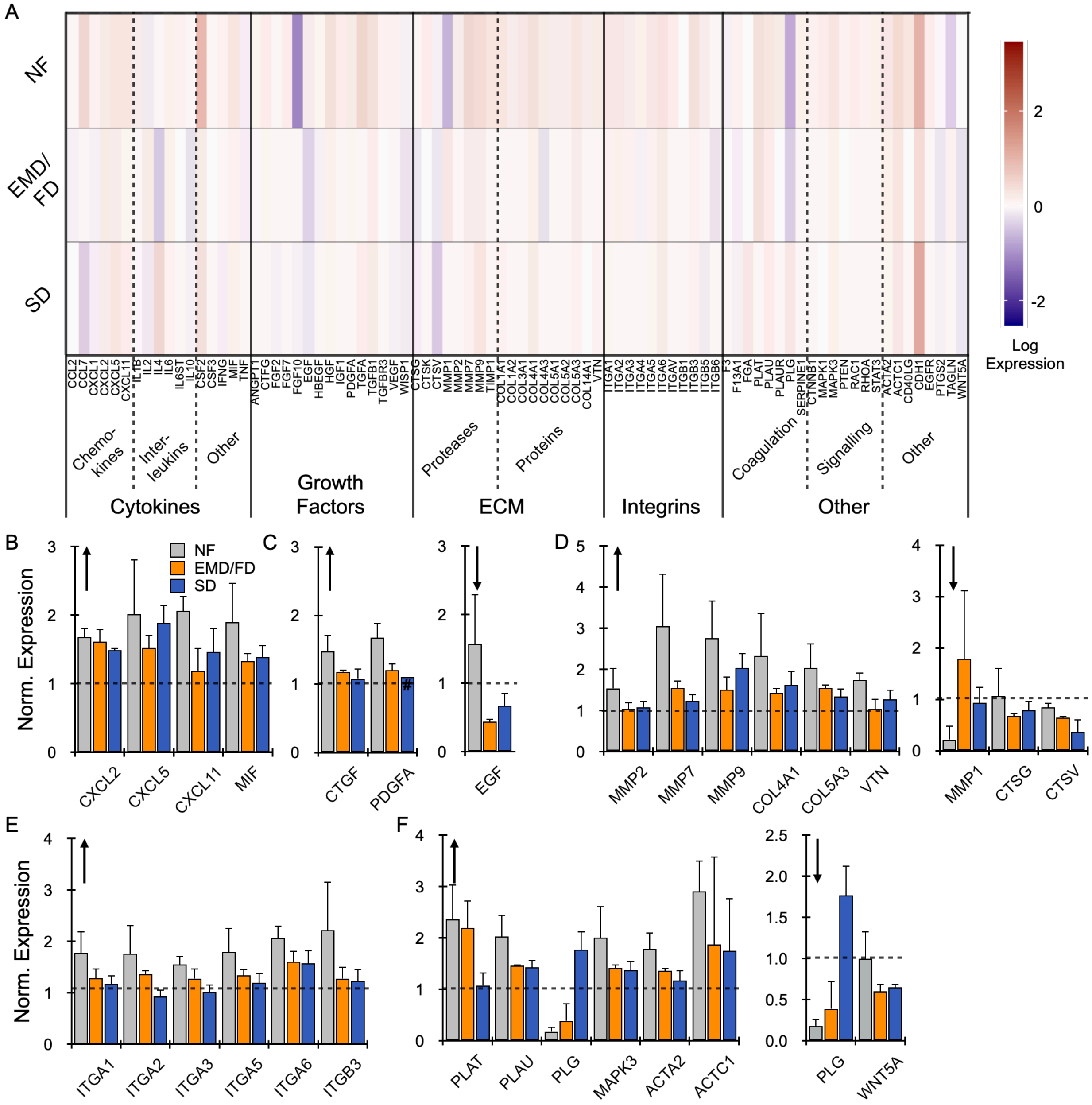
A
NF
EMD/ FD
SD
Log Expression
2
0
-2
CCL2 CCL7 CXCL1 CXCL2 CXCL5 CXCL11 IL1B IL2 IL4 IL6 IL6ST IL10 CSF2 CSF3 IFNG MIF TNF
ANGPT1 CTFG FGF2 FGF7 FGF10 EGF HBEGF HGF IGF1 PDGFA TGFA TGFB1 TGFBR3 VEGF WISP1
CTSG CTSK CTSV MMP1 MMP2 MMP7 MMP9 TIMP1 COL1A1 COL1A2 COL3A1 COL4A1 COL4A3 COL5A1 COL5A2 COL5A3 COL14A1 VTN
ITGA1 ITGA2 ITGA3 ITGA4 ITGA5 ITGA6 ITGAV ITGB1 ITGB3 ITGB5 ITGB6
F3 F13A1 FGA PLAT PLAU PLAUR PLG SERPINE1 CTNNB1 MAPK1 MAPK3 PTEN RAC1 RHOA STAT3 ACTA2 ACTC1 CD40LG CDH1 EGFR PTGS2 TAGLN WNT5A
Chemo- kines
Inter- leukins
Other
Cytokines
Growth Factors
Proteases
Proteins
ECM
Integrins
Coagulation
Signalling
Other
Other
B
C
D
E
F
Norm. Expression
NF
EMD/FD
SD
CXCL2 CXCL5 CXCL11 MIF
CTGF PDGFA
#
EGF
MMP2 MMP7 MMP9 COL4A1 COL5A3 VTN
MMP1 CTSG CTSV
ITGA1 ITGA2 ITGA3 ITGA5 ITGA6 ITGB3
PLAT PLAU PLG MAPK3 ACTA2 ACTC1
PLG WNT5A

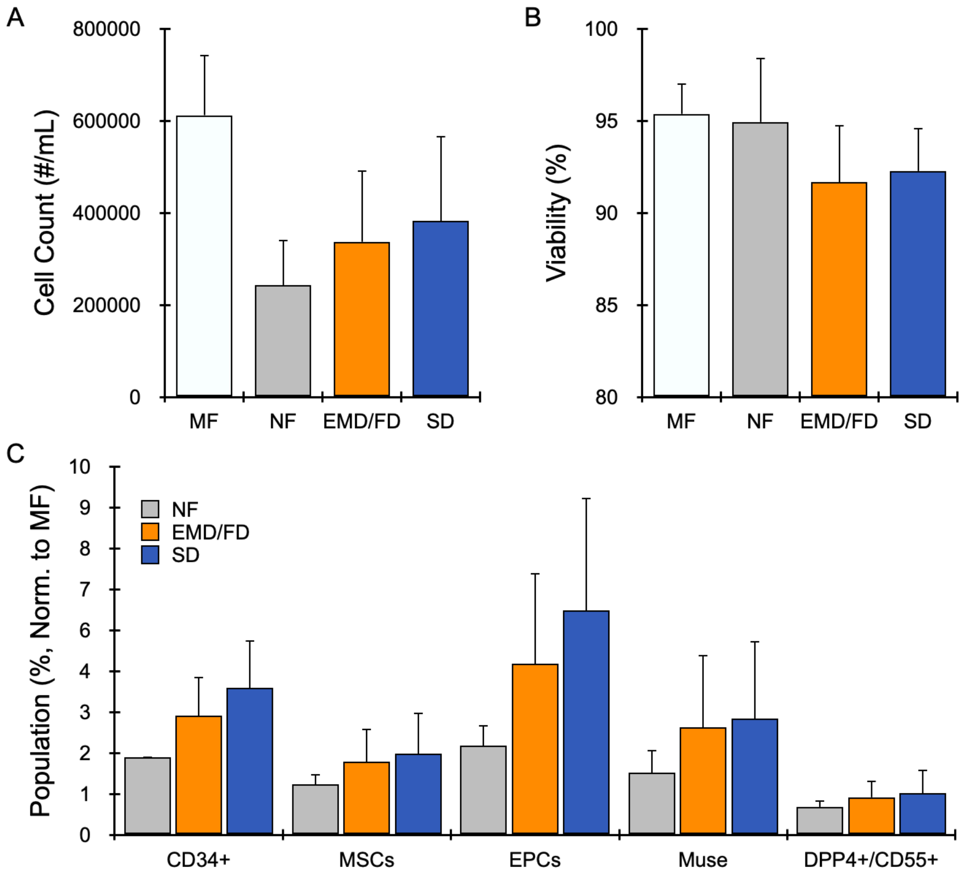

Supplemental Figure 4. Effect of cell culture. Human LA (N=3) was processed as NF, with the EMD and FD (ED/FD), or EMD/FD followed by SD at 15 mL/s. Samples were then placed in media for 24 hours. Total nucleated (A) cell count and (B) viability displayed similar trends to immediately following processing (Fig. 2). However, MF cell count decreased by >30%, while EMD/FD and SD conditions only decreased by ~20%. (C) Stem and progenitor cells remained enriched by NF and, to a greater general extent, by EMD/FD or SD. Error bars represent standard errors from at least three independent experiments.

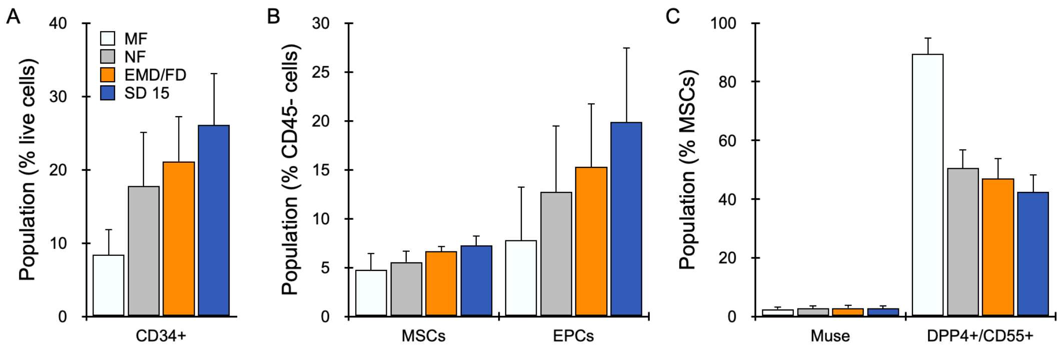

Supplemental Figure 5. Culture subpopulation results without normalization. The results from Fig. 3C in the main text are presented without normalizing to MF. Population percentages are shown separately for (A) CD34+ cells, (B) MSCs and EPCs, and (C) Muse and DPP4+/CD55+ cells. Error bars represent standard error from at least three independent experiments.

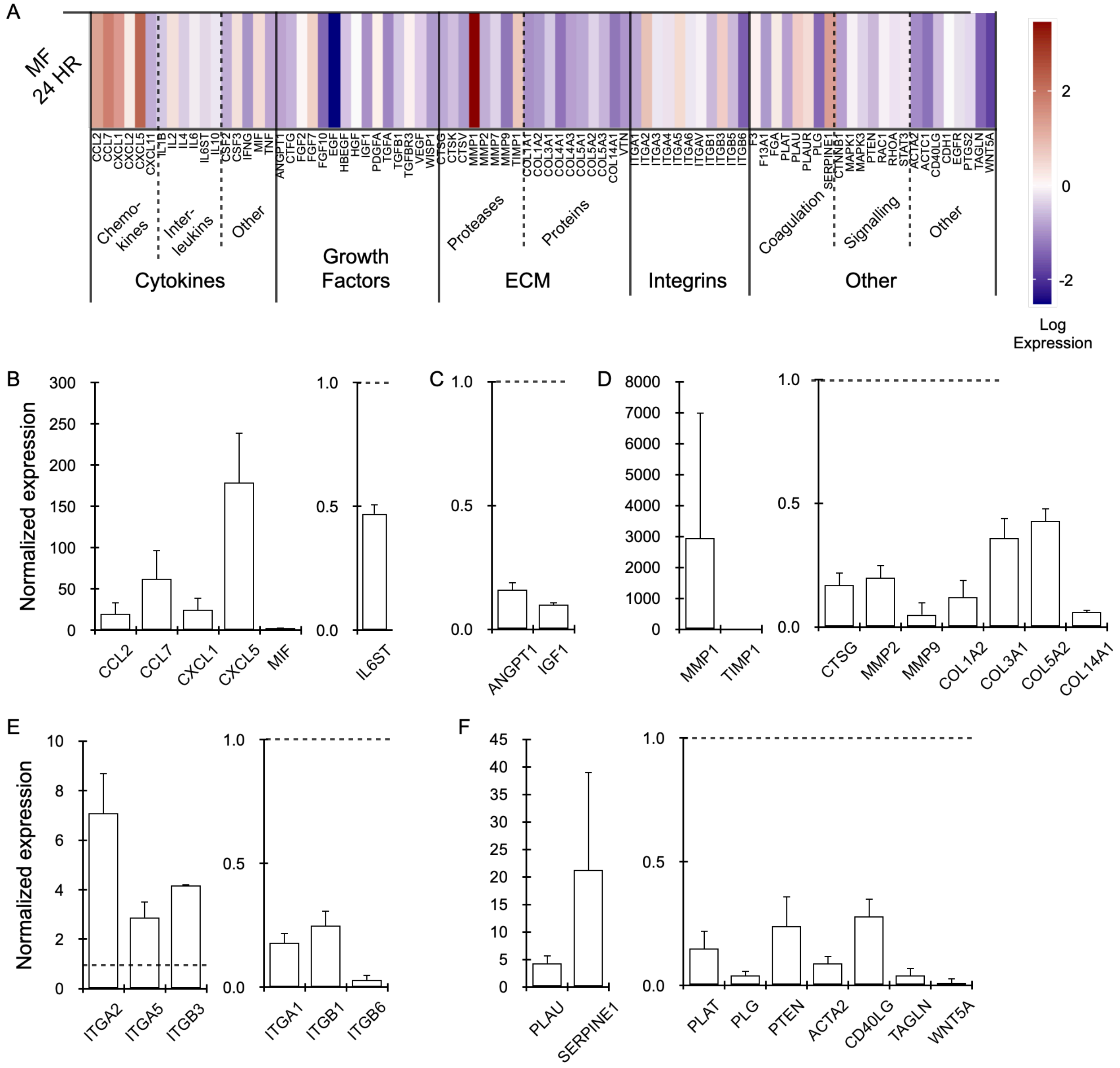

Supplemental Figure 6. Gene expression for MF immediately after processing and after 24-hour culture. Human LA (N=4) was processed as NF, with the EMD and FD (EMD/FD), or EMD/FD followed by SD at 15 mL/s. RNA was extracted and RT-qPCR was performed using a wound healing panel, and results were normalized to MF immediately after processing (value=1). (A) Heat map of all wound healing-related genes. (B-F) Results for genes in which at least one mechanical processing condition was upregulated or downregulated by >50% relative to MF. Genes are grouped as (B) cytokines, (C) growth factors, (D) extracellular matrix, (E)

integrins, and (F) other. Error bars represent standard errors from at least three independent experiments.

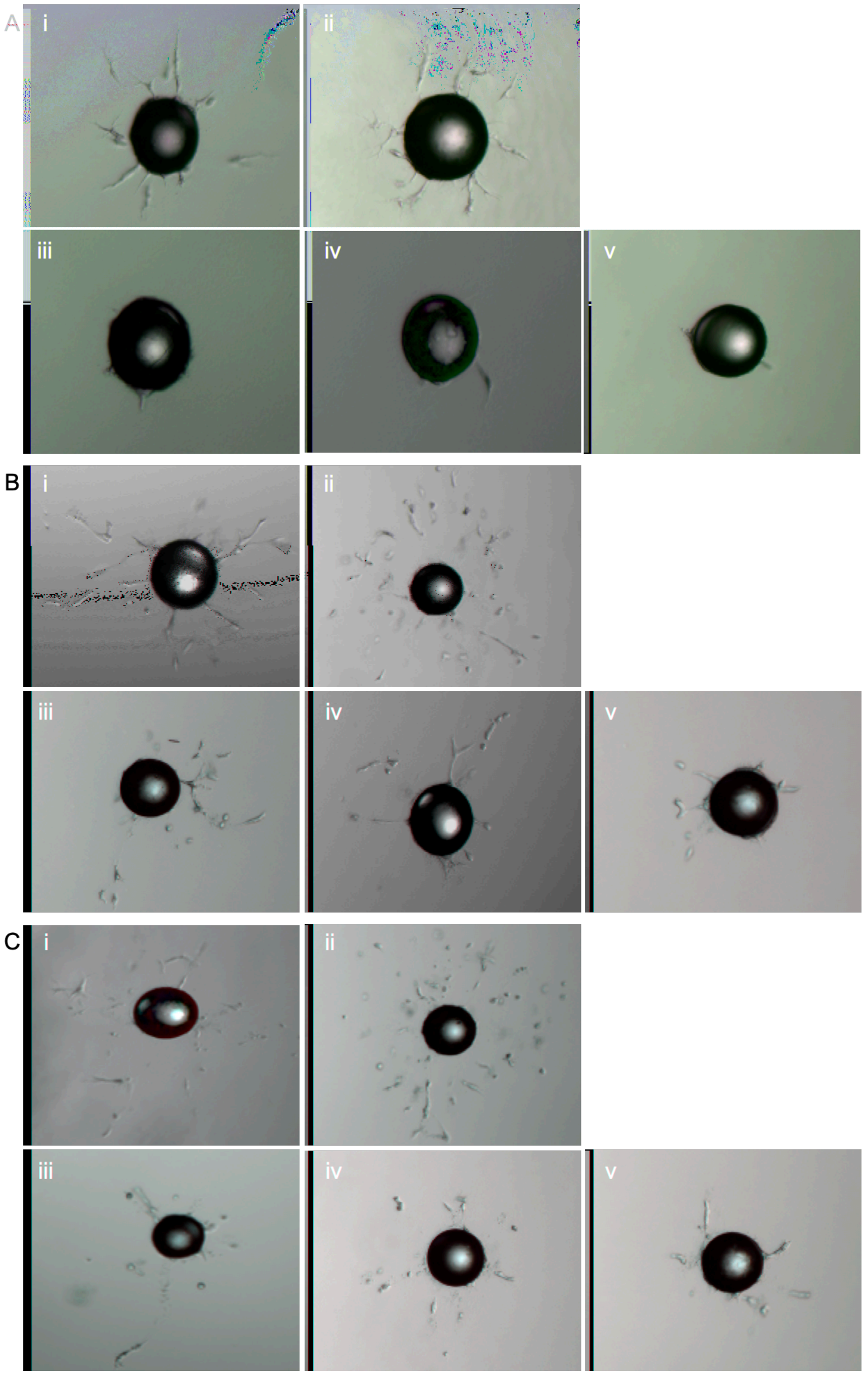

Supplemental Figure 7. Sprouting angiogenesis assay. HUVECs were coated onto collagen beds, embedded in a fibrin gel, and then co-cultured with (i) enzyme-digested MF (SVF), (ii) NF, or SVF processed with the (iii) EMD, (iv) EMD/FD, or (v) EMD/FD+SD. Representative images for each condition on days (A) 1, (B) 4, and (C) 6 of culture.
